# Avoidance of ant chemical traces by spider mites and its interpretation

**DOI:** 10.1007/s10493-022-00752-5

**Published:** 2022-10-25

**Authors:** Shuichi Yano, Mayu Konishi, Toshiharu Akino

**Affiliations:** 1 Laboratory of Ecological Information, Graduate School of Agriculture, Kyoto University, Sakyo-ku, Kyoto, 606-8502 Japan; 2 Applied Entomology Laboratory, Kyoto Institute of Technology, Saga-ippongi-cho 1, Kyoto, 616-8354 Japan

**Keywords:** Ant traces, Spider mites, *Tetranychus kanzawai*, *Tetranychus urticae*, *Pristomyrmex punctatus*, *Formica japonica*

## Abstract

Spider mites become easy prey for ants when they leave their protective webs; therefore, the ability to avoid traces of ongoing ant activity should confer a selective advantage to mites. We examined avoidance of ant traces by the spider mites *Tetranychus kanzawai* and *Tetranychus urticae*. Both mite species avoided host plant leaves with active traces of *Pristomyrmex punctatus* or *Formica japonica* ants. *Pristomyrmex punctatus* trace avoidance by *T. kanzawai* lasted for more than 1 h, but not more than 3 h. *Tetranychus kanzawai* also avoided *P. punctatus* traces on plant stems, along which the mites access leaves. Moreover, *T. kanzawai* avoided hexane extracts of *P. punctatus* or *F. japonica* applied to a filter paper pathway. This study represents the first demonstration of a repellent effect of ant chemical traces on spider mites. Considering the substantial abundance and global distribution of ants in nature, such repellent effects may help to answer the long-standing question of why only a small fraction of available plant resources is used by herbivores. Although spider mites have developed resistance against many synthetic pesticides, natural compounds that simulate ant chemical traces may repel spider mites from agricultural crops.

## Introduction

Because successful predation involves several stages—detection, identification, approach, subjugation, and consumption (Endler 1986)—the most effective antipredator defenses are those that stop predation at the earliest stages (Edmunds and Brunner 1999). Thus, some prey animals have likely developed means to detect chemical cues of predators to reduce encounters with the predators (Petranka et al. 1987; Kiesecker et al. 1996; Grostal and Dicke 1999).

Ants are generalist predators that use various semiochemicals to communicate with nestmates and conspecific foreigners (Attygalle and Morgan 1985; Hölldobler and Wilson 1990). Such chemicals within ongoing active ant territories are also detectable by their potential prey. Therefore, prey species with the ability to avoid ant chemical traces, which signal ongoing ant activity, will experience a selective advantage by reducing encounters with ants. Indeed, herbivorous beetles avoid feeding on food leaves with anal spots left by ants (Offenberg et al. 2004), and fruit flies avoid ovipositing on ant-exposed fruits (Van Mele et al. 2009). Even other predatory insects avoid ant cues. For example, ladybird beetles avoid semiochemicals left by the ants in prey (aphid) patches (Oliver et al. 2008). The beetles depredate aphids but are frequently attacked by ants tending the aphids (Oliver et al. 2008).

Spider mites in the genus *Tetranychus* feed on hundreds of wild and cultivated plant species across many plant families and are globally considered major agricultural pests (Jeppson et al. 1975; Helle and Sabelis 1985; Johnson and Lyon 1988). These mites construct three-dimensional protective webs on leaf surfaces and usually live underneath them (Saito 1983). Although mites within webs are seldom preyed upon by ants (Otsuki and Yano 2014a, b), mated female mites disperse from their webs in response to resource deterioration (Brandenburg and Kennedy 1982; Kennedy and Smitley 1985) and predator intrusion (Bernstein 1984; Grostal and Dicke 1999; Oku et al. 2004). Dispersed females settle on new hosts, and their offspring feed and grow under protective webs constructed by the females (Saito 1983). Therefore, host plant use by spider mites is practically determined by the dispersing females (Yano et al. 1998; Oku and Yano 2006). Because the mites (< 0.5 mm) are much smaller and have much less mobility compared to ants, dispersing female mites outside of the webs may exhibit low probabilities of survival when targeted by ants. Therefore, any trait that reduces encounters between female spider mites and ants should confer a selective advantage to the mites. Both *T. urticae* (Grostal and Dicke 1999; Škaloudová et al. 2007) and *Tetranychus kanzawai* (Bowler et al. 2013) exhibit the ability to detect chemical cues left by predatory mites; however, no studies have examined their abilities to detect chemical cues left by ants.

In the present study, we explored whether mated females (i.e., the dispersing stage) of *T. urticae* and *T. kanzawai* (< 0.5 mm) avoid traces of the co-occurring ants, *Pristomyrmex punctatus* (ca. 2.5 mm) and *Formica japonica* (ca. 5 mm). *Pristomyrmex punctatus* often co-occurs with *T. kanzawai* on wild plants such as bush killer, *Cayratia japonica* (Vitaceae), and has been reported to depredate *T. urticae* and *T. kanzawai* under laboratory conditions (Otsuki and Yano 2014a, b; Adachi and Yano 2017). *Formica japonica* is among the most abundant ant species in western Japan, but it does not prey upon mites presumably because mites are much smaller (S Yano unpubl.). We further examined trace avoidance characteristics using *P. punctatus* and *T. kanzawai*, which exhibit natural predator–prey interactions in the wild. In particular, chemical attributes of trace avoidance are discussed in the context of non-consumptive biocontrol of spider mites in both natural and agricultural ecosystems.

## Materials and methods

### Arthropods

In 2018, we collected > 50 adult females of *T*. *kanzawai* from trifoliate orange trees (*Poncirus trifoliata*) in Kyoto, Japan. Because *T. urticae* does not occur on wild plants in Kyoto, > 100 adult females of *T*. *urticae* were collected from chrysanthemum plants (*Chrysanthemum morifolium*, Asteraceae) in 1998 in Nara, Japan. These populations were reared on kidney bean (*Phaseolus vulgaris*, Fabaceae) leaf discs formed by pressing expanded primary leaves onto water-saturated cotton in plastic Petri dishes (90 mm diameter, 14 mm high). *Phaseolus* vulgaris is among the most suitable host plants for *T. kanzawai* (Yano et al. 2003) and *T. urticae* (Yano et al. 1998). The water-saturated cotton served as a barrier to prevent mites from escaping. The leaf discs were placed in ventilated transparent plastic containers (22 × 30 × 6 cm) maintained at 25 °C using automatically controlled air conditioners and 60% RH using automatically controlled dehumidifiers. A 16 h light (07:00–23:00):8 h dark photoperiod was maintained using timer-controlled fluorescent lamps. All the following laboratory experiments were conducted under these conditions. Each study population of spider mites was consistently maintained at > 1000 individuals. We maintained the mites for > 10 (*T. kanzawai*) and > 500 (*T. urticae*) generations prior to the experiment.

**Fig. 1:**
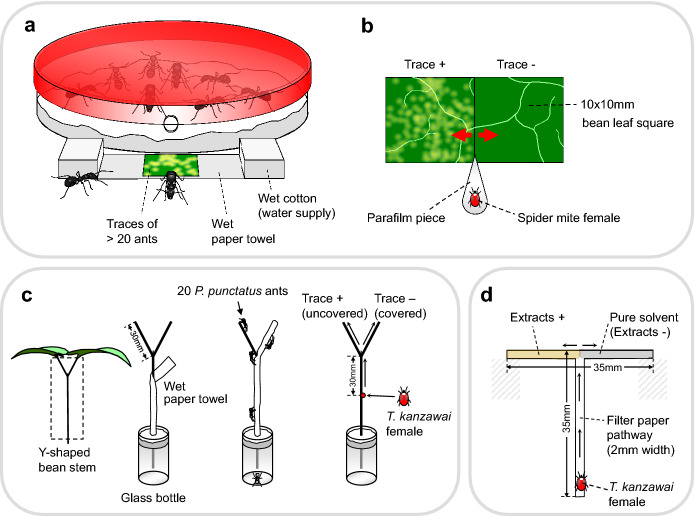
Experimental setups used to **a** introduce ant traces on a leaf square and to investigate **b** the avoidance of ant traces by spider mites on leaves, **c** the avoidance of *Pristomyrmex punctatus* traces by *Tetranychus kanzawai* on stems, and **d** the avoidance of ant trace extracts by *T. kanzawai*

We collected > 100 *P*. *punctatus* and *F*. *japonica* worker ants in Kyoto, Japan. The ants were reared in separate microcosms, each containing an artificial ant nest placed in a transparent plastic container (22 × 30 × 6 cm). A plastic Petri dish (85 mm diameter, 11 mm high) with a wet 6 mm plaster layer on the bottom and a cover painted with red pigment was used as an artificial ant nest (Fig. 1a). The ants were fed water and honey twice per week and freshly killed insects weekly as protein sources following an established protocol (Otsuki and Yano 2014a). Examples of insect prey include diamondback moth larvae (*Plutella xylostella*), termite workers (*Reticulitermes speratus*), adult vinegar flies (*Drosophila melanogaster*), and adult mosquitoes (*Aedes albopictus*). The ants were maintained under laboratory conditions described above for > 1 month before the experiment. During this period, the number of *P. punctatus* individuals increased to > 130 through worker reproduction, whereas that of *F. japonica* individuals decreased slightly from 100 due to a lack of recruitment. *Pristomyrmex punctatus* is a queenless ant species (Mizutani 1980); all workers are reproductive when young and forage outside the nest as they grow older. There have been many reports of the effects of queen absence on worker reproduction in *Formica* ants, but orphaned colonies usually continue to forage (Helanterä and Sundström 2004, 2007). Therefore, the colony fragment used in our experiments was characterized by active foraging, similar to an entire colony.

In 2020, we collected ca. 10 individual *Aphis gossypii* aphids from bush killer plants in Kyoto, Japan. The aphids were maintained on *C. japonica* leaf discs pressed onto water-saturated cotton for more than two generations under laboratory conditions described above. During the maintenance period, the aphid population increased to > 200 individuals via thelytoky.

### Avoidance of ant traces on leaf surfaces by spider mites

To examine whether spider mites avoid settling on food plant surfaces with ant traces, we conducted dual-choice experiments using paired adjacent leaf squares with and without ant traces under laboratory conditions described above. To preclude the effect of time, we conducted the following experiments within a short time period (13:30–16:30), when adult female spider mites actively disperse by walking. We used fully expanded fresh primary kidney bean leaves for the experiment. We cut a 1 × 2 cm flat leaf piece into two equal squares (1 × 1 cm) and placed one square on a piece of wet paper towel in front of the entrance to the ant nests, resembling a doormat, to introduce ant traces to the square (Fig. 1a). We introduced traces of > 20 ants to each leaf square because preliminary observations indicated that a minimum of ca. 20 ant trails were required to induce traces on all areas of the square. *Formica japonica* ants seldom left their artificial nests, which allowed for little replication. After > 20 ants had walked across the square, we transferred the square to touch the other square (without ant traces) on water-saturated cotton. We then placed a 2- to 4-day-old mated adult female of *T. urticae* or *T. kanzawai* on a pointed piece of Parafilm in contact with both leaf edges using a fine brush (Fig. 1b). We recorded the leaf square onto which the mite settled at 1 h after its introduction because preliminary observations confirmed that all females would settle on a particular leaf position within that period. Each female mite and pair of leaf squares was used only once. We used 28 replicate mites of *T. urticae* and 27 of *T. kanzawai* for *P. punctatus* traces, as well as 16 (*T. urticae*) and 12 (*T. kanzawai*) replicates for *F. japonica* traces. The data were subjected to two-tailed binomial tests with the common null hypothesis that a spider mite would settle on the two squares with equal probability (i.e., 0.5). All statistical analyses were performed using R v.3.2.2 (R Core Team 2015).

To determine whether spider mites avoid traces of species other than ants, we confined the three final instars of *A. gossypii* aphids (ca. 1 mm body length) on a 1 × 1 cm bean leaf square under laboratory conditions described above. Aphid individuals that escaped from the bean square during the 5 min introduction period were gently transferred back to the square using a fine brush. After 5 min, we transferred the square to touch the other square (without aphid traces). We then introduced an adult female of *T. urticae* (n = 35) or *T. kanzawai* (n = 36) on the leaf squares in the same manner as described above. We recorded the leaf square onto which the mite settled. The numbers were compared using binomial tests.

### Duration of *Pristomyrmex punctatus *trace avoidance by* Tetranychus kanzawai*

To examine whether the effects of ant traces on spider mite avoidance decline over time, we prepared leaf squares with *P. punctatus* traces in the same manner described above, and preserved them on water-saturated cotton for 1 h (n = 28), 3 h (n = 37), and 24 h (n = 32) under laboratory conditions described above. We then transferred the square to lie in close proximity to the other square (without ant traces) that had been preserved for the same periods of time, and compared the avoidance response of *T. kanzawai* females using binomial tests. *Tetranychus kanzawai* and *P. punctatus* were selected because only these two species exhibit natural predator–prey interactions in Kyoto.

### Avoidance of *Pristomyrmex punctatus* traces on plant stems by *Tetranychus kanzawai*

To examine whether *T. kanzawai* females avoid walking along plant stems bearing ant traces, we conducted dual choice experiments using Y-shaped kidney bean stems (Fig. 1c) under laboratory conditions described above. We cut symmetric bean plants from their bases ca. 15 days after sowing and inserted them perpendicularly into 5 mL glass bottles filled with water and wet cotton. We removed cotyledons, primary leaves, extrafloral nectaries, and buds from the stems. To induce ant traces on one branch of the Y-shaped stem, we covered the main stem and one branch with a piece of wet paper. Then, we introduced 20 ants onto the tip of the uncovered branch. All ants walked along the uncovered branch and soon escaped from the plant via the main stem covered with wet paper. Following trace induction, we carefully removed the wet paper from the plant (trace + , n = 11). To address environmental bias between covered and uncovered branches (see Results), we used Y-shaped stems without ant traces as controls (trace –, n = 28). Then we introduced a *T. kanzawai* female onto a release point 3 cm below the bifurcation of each Y-shaped stem using a fine brush (Fig. 1c). We recorded the branch along which the female walked to the far end. Each female mite and each Y-shaped stem was used only once. The proportions of females that walked along the uncovered branch were compared between ‘trace + ’ and ‘trace –’ plants using Fisher’s exact test.

### Avoidance of ant trace extracts by *Tetranychus kanzawai*

To extract chemical traces of ants, we introduced 20 *P. punctatus* ants to a glass Petri dish (45 mm diameter, 16 mm high) under laboratory conditions described above and covered the dish with a glass slide. We introduced the ants carefully to ensure that they would not be excited by the movement. After 1 h, we removed all ants and washed the inside bottom of the dish with 500 µL of hexane. We introduced 10 *F. japonica* ants to a glass Petri dish (56 mm diameter, 3 cm high) as described above. The interior wall of the dish was coated with fluon (AGC Chemicals, Tokyo, Japan) to prevent the ants from escaping. After 2 h, we removed all ants and washed the inside bottom of the dish with 500 µL of hexane. For each ant species, we replicated the procedure 20 × using different individuals to combine all extracts and to acquire enough extract for the following experiment. For the control solvent, we poured the same amount of hexane into dishes left for 1 or 2 h without ants, and then consolidated the solvent as described above.

To examine avoidance of ant trace extracts by *T. kanzawai* females, we conducted dual-choice experiments using T-shaped pathways of filter paper (35 × 35 mm, 2 mm wide; Fig. 1d) under laboratory conditions described above. Using disposable micropipettes (Drummond Scientific, Broomall, PA, USA), we applied 0.175 ant equivalent (i.e., 5 µL) of hexane extract to an alternately selected branch (17.5 mm long) of each pathway (i.e., 0.01 ant equivalent/mm), with the control hexane applied to the other branch. The extract dose of 0.01 ant equivalent/mm reflects the traces of 20 ants during a period of 5 min, which approximately corresponds to the number of ants that left trails in previous experiments. We applied each solution dropwise at the junction point to minimize mixing. After evaporating the solvent from those pathways, we perpendicularly suspended them (Fig. 1d) and introduced a female adult at 2 days post-maturation onto the bottom of each pathway using a fine brush and recorded the branch along which the female first walked to the far end. Each female mite and T-shaped filter paper were used only once, with 46 replicates for *P. punctatus* and 44 replicates for *F. japonica*. Each female mite made a choice within 10 min. Avoidance was analyzed using a binomial test.

## Results

### Avoidance of ant traces on leaf surfaces by spider mites

**Fig. 2:**
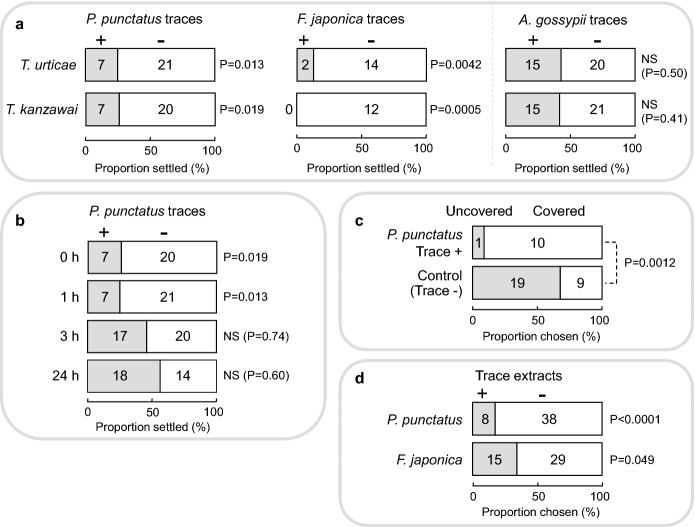
**a** Avoidance of ant (﻿*Pristomyrmex punctatus*, *Formica japonica*) and aphid (*Aphis gossypii*) traces on leaf surfaces by *Tetranychus urticae* and *T. kanzawai*. Both spider mite species avoided ant traces, but not aphid traces (binomial test). **b** Duration of *P. punctatus* trace avoidance on leaf surfaces by *T. kanzawai*. Spider mites avoided 0- and 1 h-old traces but did not avoid 3- and 24 h-old traces (binomial test). The data for 0 h were identical to those presented in (**a**). **c** Avoidance of *P. punctatus* traces on plant stems by *T. kanzawai*. Although spider mites tended to avoid branches that had previously been covered with wet paper (trace –; binomial test: *P* = 0.087), fewer mites walked along uncovered branches with ant traces than along branches without traces (Fisher’s exact test), indicating that the mites avoided ant traces on plant stems. **d** Avoidance of *P. punctatus* and *F. japonica* trace extracts applied to filter paper pathways by *T. kanzawai*. Spider mites avoided the trace extracts of both ant species (binomial test)

Significantly fewer *T. urticae* females settled onto leaf squares with traces of either *P. punctatus* or *F. japonica* ants (Fig. 2a). Similarly, significantly fewer *T. kanzawai* females settled onto leaf squares with traces of *P. punctatus* or *F. japonica* ants (Fig. 2a). In contrast, the number of *T. urticae* and *T. kanzawai* females settled did not differ significantly between control squares and squares with traces of *A. gossypii* aphids (Fig. 2a). These results indicate that both mite species avoided ant traces but not aphid traces.

### Duration of *Pristomyrmex punctatus* trace avoidance by *Tetranychus kanzawai*

*Tetranychus kanzawai* females significantly avoided 0- and 1-h-old traces of *P. punctatus*, but did not avoid 3- and 24-h-old traces (Fig. 2b), suggesting that the avoidance of *P. punctatus* traces by *T. kanzawai* lasted for more than 1 h but not for more than 3 h.

### Avoidance of *Pristomyrmex punctatus* traces on plant stems by *Tetranychus kanzawai*

In the absence of ant traces (control), *T. kanzawai* females tended to avoid branches that had previously been covered with wet paper (binomial test: *P* = 0.087). However, significantly fewer mites walked along uncovered branches with ant traces than along branches without traces (Fig. 2c), indicating that the mites avoided walking along ant traces on plant stems.

### Avoidance of ant trace extracts by *Tetranychus kanzawai*

Significantly fewer *T. kanzawai* females walked along the branch applied with trace extracts of *P. punctatus* or *F. japonica* (Fig. 2d), indicating that the mites avoided ant trace extracts.

## Discussion

This study is the first to demonstrate that spider mite females, which mainly determine mite host use (Yano et al. 1998; Oku and Yano 2006), avoided ant traces. The avoidance of ant traces, which decline over time, diverts spider mites from active ant territories, thereby reducing their predation by ants. However, the cost of ant trace avoidance by spider mites on food plants may be considerable under certain conditions. The reason that *T. kanzawai* mites tended to avoid branches that had previously been covered with wet paper remains unknown; however, the mites clearly avoided walking along ant traces on feed plant stems. Because most plant leaves are hierarchically connected by stems, spider mites that avoid walking along ant traces on a plant stem must abandon all food leaves on that stem. The avoidance of ant traces on stems by female mites also reduces available food resources for their offspring.

Spider mites avoided traces of *P. punctatus* ants that depredate mites (Otsuki and Yano 2014a, b; Adachi and Yano 2017) and those of *F. japonic*a ants, which do not appear to prey upon them (S Yano, unpubl.). By contrast, female mites did not avoid aphid traces. Previous studies also reported that *T. urticae* females do not avoid traces of cowpea aphids (Adachi and Yano 2017), fungivorous mites, or pollen-feeder mites (Grostal and Dicke 2000). Therefore, it is unlikely that spider mites avoid any trace left on leaf surfaces.

This study is also the first to reveal that ant trace avoidance by *T. kanzawai* is associated with chemical residues in the traces, which may explain the decline in ant trace avoidance by spider mites over time and the apparent non-specificity of this avoidance to predatory ant species. It may be that spider mites cannot develop species-specific avoidance strategies against all predator species due to their limited cognitive ability. This hypothesis coincides with the fact that *T. urticae* avoids traces of both enemy and non-enemy predatory mite species (Grostal and Dicke 2000). However, spider mites are expected to strongly benefit from avoiding the traces of many ant species because ant species other than *P. punctatus* also depredate spider mites (Haney et al. 1987; Osborne et al. 1995) and many ant species whose workers are similar in size to those of *P. punctatus* prey upon spider mites under laboratory conditions (S Yano, unpubl.). Because ants are generalist predators that consume many arthropod prey species, the need to avoid ant trace chemicals is not restricted to spider mites. Indeed, some herbivorous (Offenberg et al. 2004; Van Mele et al. 2009) and predatory (Oliver et al. 2008) insects reportedly avoid ant cues left in their habitats. Additional studies are needed to clarify how often ant traces and their chemical extracts are avoided by pest arthropods other than spider mites.

Predators reduce prey population densities through consumption and by driving prey to adopt defenses (Werner and Peacor 2003; Preisser et al. 2005) that are costly due to reduced foraging (Morrison 1999; Downes 2001), enhanced predation risk (Losey and Denno 1998; Sih et al. 1998; Otsuki and Yano 2014a) or exposure to abiotic stressors (Hirayama and Kasuya 2014; Okada and Yano 2021). The non-consumptive effects of predators on prey are sometimes comparable in strength to the effects of direct consumption (Lima 1998; Morrison 1999; Bolker et al. 2003; Creel and Christianson 2008; Okada and Yano 2021). Considering the vast abundance of ants in nature (Hölldobler and Wilson 1994) and that their repellent traces do not immediately decline, prey arthropods that avoid ant traces may have far less flexibility in colonizing and moving onto their host plants than previously expected. Such non-consumptive control of prey by ant traces may partly explain why only a small fraction of available plant resources without effective defenses is used by herbivores (Hairston et al. 1960; Strong and Lawton 1984).

It is possible that spider mites avoid substances that are commonly contained in the traces of many ant species. Identifying the compounds responsible for avoidance of ant traces by spider mites is especially important for potential applications. Ant chemical traces are widely distributed in the wild, even on the surfaces of agricultural products. Therefore, if the chemical substances responsible for avoidance are known, it would be possible to manufacture spider mite repellents that simulate ant traces, which are apparently harmless to humans. Although spider mites have developed resistance to many synthetic pesticides (Mayank 2020), they would not easily develop ‘resistance’ against repellents that simulate ant traces, as the avoidance mechanism is seemingly based on natural prey-predator interactions. That is, spider mites that do not avoid ant trace chemicals are subjected to higher predation pressure from ants.

## Data Availability

All data can be found in the figures.

## References

[CR1] Adachi M, Yano S (2017). Ant-mediated indirect negative effects of aphids on spider mites living on the same plant. Exp Appl Acarol.

[CR2] Attygalle AB, Morgan ED (1985). Ant trail pheromones. Adv Insect Physiol.

[CR3] Bernstein C (1984). Prey and predator emigration responses in the acarine system *Tetranychus urticae*-*Phytoseiulus persimilis*. Oecologia.

[CR4] Bolker B, Holyoak M, Křivan V, Rowe L, Schmitz O (2003). Connecting theoretical and empirical studies of trait-mediated interactions. Ecology.

[CR5] Bowler DE, Yano S, Amano H (2013). The non-consumptive effects of a predator on spider mites depend on predator density. J Zool.

[CR6] Brandenburg RL, Kennedy GG (1982). Intercrop relationships and spider mite dispersal in a corn/peanut agroecosystem. Entomol Exp Appl.

[CR7] Creel S, Christianson D (2008). Relationships between direct predation and risk effects. Trends Ecol Evol.

[CR8] Downes S (2001). Trading heat and food for safety: costs of predator avoidance in a lizard. Ecology.

[CR9] Edmunds M, Brunner D, Prete FR, Wells H, Wells PH, Hurd LE (1999). 13. Ethology of defenses against predators. The praying Mantids.

[CR10] Endler JA (1986). Natural selection in the wild.

[CR11] Grostal P, Dicke M (1999). Direct and indirect cues of predation risk influence behavior and reproduction of prey: a case for acarine interactions. Behav Ecol.

[CR12] Grostal P, Dicke M (2000). Recognising one’s enemies: a functional approach to risk assessment by prey. Behav Ecol Sociobiol.

[CR13] Hairston NG, Smith FE, Slobdkin LB (1960). Community structure, population control, and competition. Am Nat.

[CR14] Haney PB, Luck RF, Moreno DS (1987). Increases in densities of the citrus red mite, *Panonychus citri* (Acarina: Tetranychidae), in association with the argentine ant, *Iridomyrmex humilis* (Hymenoptera: Formicidae), in southern California citrus. Entomophaga.

[CR15] Helanterä H, Sundström L (2004). Worker reproduction in the ant *Formica fusca*. J Evol Biol.

[CR16] Helanterä H, Sundström L (2007). Worker reproduction in *Formica* ants. Am Nat.

[CR17] Helle W, Sabelis MW (1985). Spider mites: their biology, natural enemies and control (vol 1A).

[CR18] Hirayama H, Kasuya E (2014). Potential costs of selecting good sites for offspring: increased risk of drowning and negative effects on egg production. Ethology.

[CR19] Hölldobler B, Wilson EO (1990). The Ants.

[CR20] Hölldobler B, Wilson EO (1994). Journey to the ants: a story of scientific exploration.

[CR21] Jeppson LR, Keifer HH, Baker TW (1975). Mites injurious to economic plants.

[CR22] Johnson WT, Lyon HH (1988). Insects that feed on trees and shrubs.

[CR23] Kennedy GG, Smitley DR, Helle W, Sabelis MW (1985). Dispersal. Spider mites: their biology, natural enemies and control.

[CR24] Kiesecker JM, Chivers DP, Blaustein AR (1996). The use of chemical cues in predator recognition by western toad tadpoles. Anim Behav.

[CR25] Lima SL (1998). Nonlethal effects in the ecology of predator-prey interactions. Bioscience.

[CR26] Losey JE, Denno RF (1998). Positive predator–predator interactions: enhanced predation rates and synergistic suppression of aphid populations. Ecology.

[CR27] Mayank C (2020) Recent trends in insect pest management. 2 Chief Editor.

[CR28] Mizutani A (1980). Preliminary report on worker reproduction in the ant *Pristomyrmex punctatus* Mayr. Kontyu.

[CR29] Morrison L (1999). Indirect effects of phorid fly parasitoids on the mechanisms of interspecific competition among ants. Oecologia.

[CR30] Offenberg J, Nielsen MG, Havanon MDJS, Aksornkoae S (2004). Evidence that insect herbivores are deterred by ant pheromones. Proc R Soc Lond B (suppl).

[CR31] Okada S, Yano S (2021). Oviposition-site shift in phytophagous mites reflects a trade-off between predator avoidance and rainstorm resistance. Biol Lett.

[CR32] Oku K, Yano S (2006). Host plant acceptance of the phytophagous mite *Tetranychus kanzawai* Kishida is affected by availability of refuge on the leaf surface. Ecol Res.

[CR33] Oku K, Yano S, Takafuji A (2004). Nonlethal indirect effects of a native predatory mite, *Amblyseius womersleyi* Schicha (Acari: Phytoseiidae), on the phytophagous mite *Tetranychus kanzawai* Kishida (Acari: Tetranychidae). J Ethol.

[CR34] Oliver TH, Jones J, Cook JM, Leather SR (2008). Avoidance responses of aphidophagous ladybird, *Adalia bipunctata*, to aphid-tending ants. Ecol Entomol.

[CR35] Osborne LS, Pena JE, Oi DH (1995). Predation by *Tapinoma melanocephalum* (Hymenoptera: Formicidae) on twospotted spider mites (Acari: Tetranychidae) in Florida greenhouses. Fla Entomol.

[CR36] Otsuki H, Yano S (2014). Functionally different predators break down antipredator defenses of spider mites. Entomol Exp Appl.

[CR37] Otsuki H, Yano S (2014). Potential lethal and non-lethal effects of predators on dispersal of spider mites. Exp Appl Acarol.

[CR38] Petranka JW, Kats LB, Sih A (1987). Predator-prey interactions among fish and larval amphibians: use of chemical cues to detect predatory fish. Anim Behav.

[CR39] Preisser EL, Bolnick DL, Benard MF (2005). Scared to death? The effects of intimidation and consumption in predator–prey interactions. Ecology.

[CR40] R Core Team (2015). R: A language and environment for statistical computing (v.3.2.2).

[CR41] Saito Y (1983). The concept of “life types” in Tetranychinae. An attempt to classify the spinning behaviour of Tetranychinae. Acarologia.

[CR42] Sih A, Englund G, Wooster D (1998). Emergent impacts of multiple predators on prey. Trends Ecol Evol.

[CR43] Škaloudová B, Zemek R, Křivan V (2007). The effect of predation risk on an acarine system. Anim Behav.

[CR44] Strong DR, Lawton JH, Southwood TRE (1984). Insects on plants. Community patterns and mechanism.

[CR45] Van Mele P, Vayssieres J, Adandonon A, Shinzogan A (2009). Ant cues affect the oviposition behavior of fruit flies (Diptera: Tephritidae) in Africa. Physiol Entomol.

[CR46] Werner EE, Peacor SD (2003). A review of trait-mediated indirect interactions in ecological communities. Ecology.

[CR47] Yano S, Wakabayashi M, Takabayashi J, Takafuji A (1998). Factors determining the host plant range of the phytophagous mite, *Tetranychus urticae* (Acari: Tetranychidae): a method for quantifying host plant acceptance. Exp Appl Acarol.

[CR48] Yano S, Kanaya M, Takafuji A (2003). Genetic basis of color variation in leaf scars induced by the Kanzawa spider mite. Entomol Exp Appl.

